# Protection status, human disturbance, snow cover and trapping drive density of a declining wolverine population in the Canadian Rocky Mountains

**DOI:** 10.1038/s41598-022-21499-4

**Published:** 2022-10-24

**Authors:** Mirjam Barrueto, Anne Forshner, Jesse Whittington, Anthony P. Clevenger, Marco Musiani

**Affiliations:** 1 Department of Biological Sciences, University of Calgary, 507 Campus Dr NW, Calgary, AB T2N 4V8 Canada; 2 Parks Canada, Banff, Yoho and Kootenay National Parks, PO Box 213, Lake Louise, AB T0L1E0 Canada; 3 Parks Canada, Banff National Park Resource Conservation, PO Box 900, Banff, AB T1L 1K2 Canada; 4 Western Transportation Institute, Montana State University, P.O. Box 174250, Bozeman, MT 59717-4250 USA; 5 Dipartimento Scienze Biologiche Geologiche Ambientali, BiGeA, Università di Bologna, Bologna, Italy

**Keywords:** Conservation biology, Population dynamics, Statistical methods, Biodiversity

## Abstract

Protected areas are important in species conservation, but high rates of human-caused mortality outside their borders and increasing popularity for recreation can negatively affect wildlife populations. We quantified wolverine (*Gulo gulo*) population trends from 2011 to 2020 in > 14,000 km^2^ protected and non-protected habitat in southwestern Canada. We conducted wolverine and multi-species surveys using non-invasive DNA and remote camera-based methods. We developed Bayesian integrated models combining spatial capture-recapture data of marked and unmarked individuals with occupancy data. Wolverine density and occupancy declined by 39%, with an annual population growth rate of 0.925. Density within protected areas was 3 times higher than outside and declined between 2011 (3.6 wolverines/1000 km^2^) and 2020 (2.1 wolverines/1000 km^2^). Wolverine density and detection probability increased with snow cover and decreased near development. Detection probability also decreased with human recreational activity. The annual harvest rate of ≥ 13% was above the maximum sustainable rate. We conclude that humans negatively affected the population through direct mortality, sub-lethal effects and habitat impacts. Our study exemplifies the need to monitor population trends for species at risk—within and between protected areas—as steep declines can occur unnoticed if key conservation concerns are not identified and addressed.

## Introduction

Protected areas play an important role in species conservation worldwide^1,2^. Their biophysical attributes, human activities within, and interplay with surrounding unprotected lands influence population dynamics and viability of the species they aim to conserve^3^. Elevated rates of human-caused mortality outside protected area boundaries can negatively affect species’ populations within protected areas, resulting in edge effects^4,5^. Yet, for reasons including reserve location, funding allocation by governments and management objectives, protected areas worldwide and in Canada are often not effectively protecting biodiversity or target species^1,6,7^. For example, protected areas are increasingly popular for outdoor recreation^8^, with implications for wildlife^9,10^. Alterations made because of recreational activities can impact species directly through habitat loss^11,12^. Recreating humans may also be perceived as a threat by wildlife^13^, thus impacting them indirectly. If animals become stressed or avoid otherwise suitable habitat, significant costs to individuals may be incurred^14,15^. If survival and reproduction are affected, population-level effects are possible, including species’ declines^10,15^.

Monitoring focal species populations over time to understand population trends and to evaluate impacts of management actions is needed for sound wildlife management within and outside protected areas^16^. For wide-ranging, wary species occurring at low densities, monitoring has long been a challenge due to budget and logistical constraints. Non-invasive sampling methods based on individual identification from DNA samples or remote cameras enable comparatively cost-effective sampling across large areas to estimate population size^17^. With camera-based methods entire suites of species can be simultaneously monitored^18,19^. Furthermore, integrated biostatistical models use multiple data sources to inform common parameters, which expands the number of parameters that can be estimated, increases the precision of estimates, reduces bias, and can bridge temporal sampling gaps^20,21^. Finally, consistency in sampling methodology is essential to achieve comparable estimates^22^.

Large carnivore populations worldwide have substantially declined due to human impacts^23^, and protected areas are considered crucial to maintaining their populations^24^. Wolverines (*Gulo gulo*) are circumboreal apex predators with large home ranges, extremely low densities and slow life histories^25^, which have undergone extensive range contractions worldwide but remained little studied until relatively recently (reviewed in Fisher et al.^26^). Conservation risks include but are not limited to vulnerability to overharvest^27,28^, habitat loss^29^, and disturbances from recreation^30^. Due to their high detectability at baited stations and their unique fur markings, wolverines are well-suited for non-invasive DNA and remote camera monitoring techniques^31,32^. Yet, wolverine population inventories have only been conducted in small pockets within their worldwide and North American range^26^, and with the exception of Norway and Sweden^33,34^, contemporary population density trends are unknown, which complicates development of appropriate management actions.

**Figure 1:**
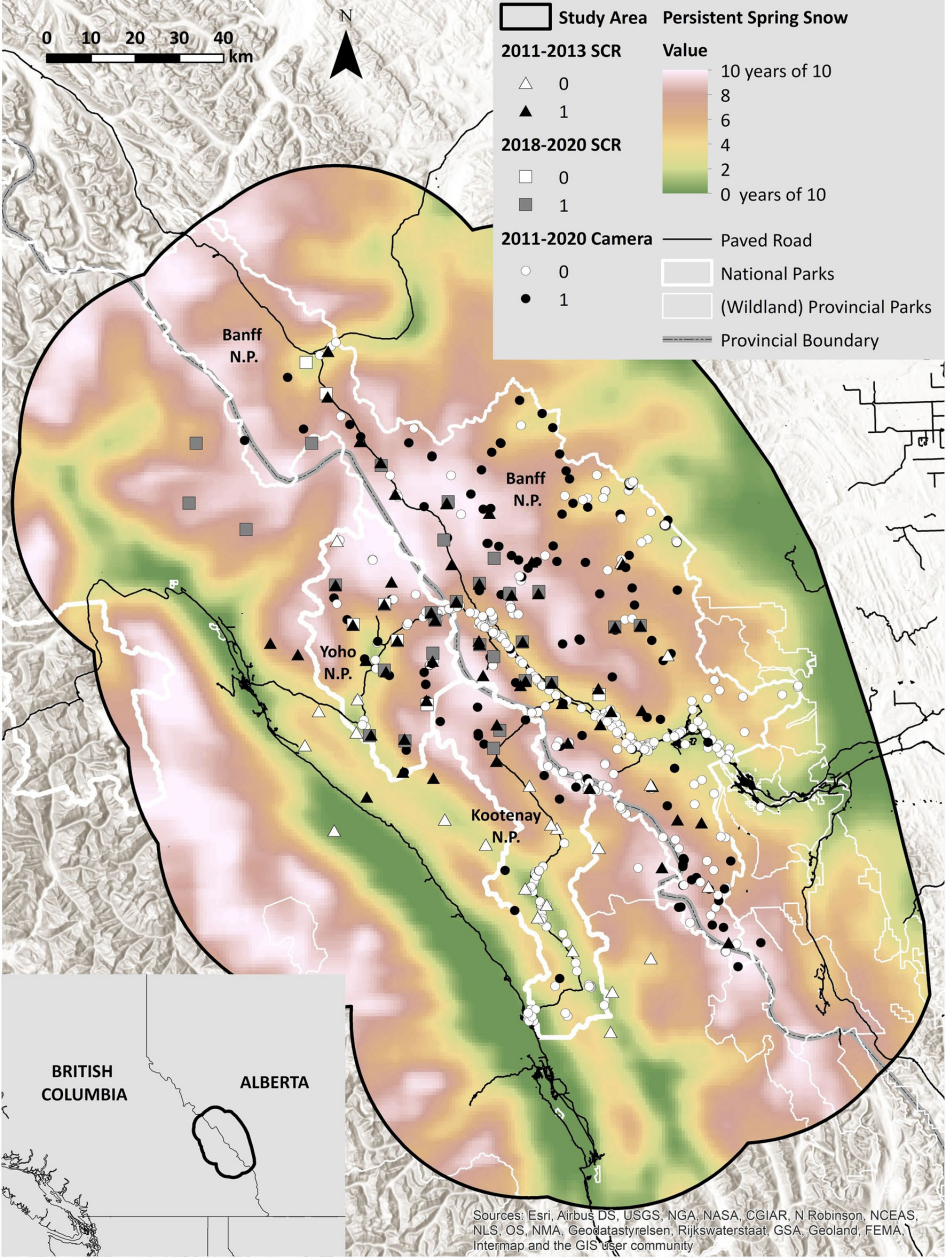
The study area, outlined in black, of a 10-year study on wolverine (*Gulo gulo*), conducted between 2011 and 2020 in southwestern Canada. Study area size was 30,689 km^2^. Symbols depict wolverine sampling stations, where grey or black symbols mean that wolverines were detected at least once. White means that no wolverines were detected. Sampling sites for the three field studies are depicted with triangles (“2011–2013 SCR”), squares (“2018–2020 SCR”), and circles (“2011–2020 Camera”). Background colors indicate the number of years out of 10 a pixel had complete spring snow cover between 2010 and 2020, using a 10 km moving average. National parks are outlined in white (thick line), provincial protected areas in white (thin line), and paved roads as thin black lines. Sources for the base map include Esri, Airbus DS, USGS, NGA, Nasa, CGIAR, N Robinson, NCEAS, NLS, OS, NMA, Geodatastyrelsen, Rijkswaterstaat, GSA, Geoland, FEMA, Intermap, and the GIS user community. This map contains information licensed under the Open Government Licence—British Columbia (https://www2.gov.bc.ca/gov/content/data/open-data/open-government-licence-bc), the Open Government Licence—Alberta (https://open.alberta.ca/licence) and the Open Government Licence—Canada (https://open.canada.ca/en/open-government-licence-canada) and was created in ArcMap 10.7.1 (https://support.esri.com/en/products/desktop/arcgis-desktop/arcmap/10-7).

Between 2011 and 2020, we conducted two targeted, non-invasive, baited wolverine density surveys in southwestern Canada (Fig. 1). The study area centred on three national parks, which we refer to as “protected areas”, with reference to both the protection status of the land and protection status of wolverine from harvest within the parks. In the adjacent “unprotected areas” within our study area, which included land used for forestry and other industries but also several provincial parks, wolverine harvest was allowed during our study. Historically, wolverine culling as part of predator control efforts also occurred within the protected areas, but ended in 1959^35^. Studies that included data from the earlier of our two surveys (2011–2013) showed that there was evidence of an edge effect within the protected areas, whereas density inside parks declined towards the park boundaries^36^. Wolverine occupancy was twice as high within the protected areas compared to outside, and a model which included natural landcover, linear industrial features, persistent spring snow cover and mesocarnivore occurrence best explained wolverine distribution, but that study did not assess the effects of wolverine harvest^37^. Regionally, however, protected areas were the strongholds of the species, and wolverine harvest was unsustainable^28^.

Concurrent to the two targeted and baited wolverine surveys (2011–2013 and 2018–2020), we carried out non-targeted, unbaited camera surveys (2011–2020) designed to monitor large mammals, including wolverine^18,38^. Using the spatial capture-recapture (SCR) framework^17^, we combined SCR data sets form the targeted surveys with occupancy data derived from the camera surveys (Table 1) and developed novel integrated spatially explicit population models^21,39^. As the abundance measure, we used density (unit: individuals/1000 km^2^), which enables direct comparisons over time and space.

**Table 1:** Data used in a 10-year study on wolverines (*Gulo gulo*), conducted between 2011 and 2020 in the Canadian Rocky Mountains in southern Canada.

Data	Individual identification	Detection (y = 0 or 1) of any wolverine	Unidentifiable individuals
2011–2013 SCR	DNA	Images	–
2018–2020 SCR	DNA; Images of chest patterns	–	Poor quality DNA; poor quality images
2011–2020 Camera	–	Images	–

Data for integrated spatial capture–recapture (SCR) models included individuals with known identity (2011–2013 and 2018–2020 SCR), detection data for any wolverine (2011–2013 SCR and 2011–2020 remote camera), and individuals that were unknown and could not be classified to individual (2018–2020 SCR). Method of classification to individual changed from DNA only in 2011–2013 to the combination of DNA and remote camera images (2018–2020).

Our objectives were to estimate 10-year wolverine population trends, mortality rates from trapping, and the effects of recreational activities and human development on wolverine density and detection probability. We guided our analysis with three non-mutually exclusive hypotheses: (1) Regionally unsustainable wolverine harvest rates^28^ led to declining wolverine densities in the unprotected parts of our study area. We expected to see higher wolverine densities within protected areas than outside, and declining densities outside protected areas; (2) Protected areas fulfilled their role as harvest refugia^40,41^. We expected to find that wolverine density within the national parks remained stable between 2011 and 2020; (3) Wolverines perceived human presence as a threat^30^, such that recreation and human development^42^ caused measurable spatial avoidance by wolverines. We expected that wolverine detection was lower in areas with higher numbers of humans on camera, and that density was lower in areas with high levels of development.

## Results

### Individuals and sample sizes

We detected 41 individuals during the 2011–2013 SCR sampling. We identified wolverine at 57 (71%) of the 80 sampling sites. We sampled 346 occasions across the three years with three occasions per year (occasion duration mean = 30.9 days, SD = 3.6). Remote cameras at SCR sites detected wolverine on 219 occasions. We extracted DNA from hair deposited when wolverine were detected by camera^43^. Successful DNA extraction and genotyping of hair led to a total of 190 identifiable individual detections. In this survey, we did not classify detections as unidentifiable, as we could not reliably link individual photo detections with DNA profiles (Table 1). We detected individuals a median of 4 times and at a median of three sites per animal. 35 of the 41 individuals (85%) were detected more than once, and 35 individuals were detected at multiple sites.

During the 2018–2020 SCR sampling, we detected 21 individuals. We identified wolverine at 28 (78%) of the 36 sampled sites. We sampled 202 occasions across the 3 years with up to four occasions per year (occasion duration mean = 30.8, SD = 5.5). With a total of 123 identifiable detections, we detected individuals a median of 3 times and a median of 3 sites per animal. 17 of the 21 individuals (81%) were detected multiple times and 16 (76%) were detected at multiple sites. Remote cameras at the sites detected unidentifiable (no DNA or chest pattern obtained; Table 1) wolverine on 40 occasions.

Together, the two surveys identified 59 animals (25 females, 32 males, 2 of unknown sex). Most wolverines were detected in > 1 year, and we detected four individuals in 2018 that were first detected in 2010 (1 female, 1 male), 2012 (1 male) or 2014 (1 female). When selecting the same sampling area (the 2011–2013 survey area was larger), the 2011–2013 survey detected 33 individuals, and the 2018–2020 survey detected 19.

From 2011 to 2020, remote cameras (without bait or lure) sampled 558 sites within Banff, Kootenay, and Yoho National Parks. Cameras detected wolverine on 176 of the 2206 two-week occasions. 437 (78%) of the cameras operated for more than one winter (median = 3 years, range = 1 to 10 years). We detected wolverine at 139 (25%) of the sites.

### Density and occupancy results

**Figure 2:**
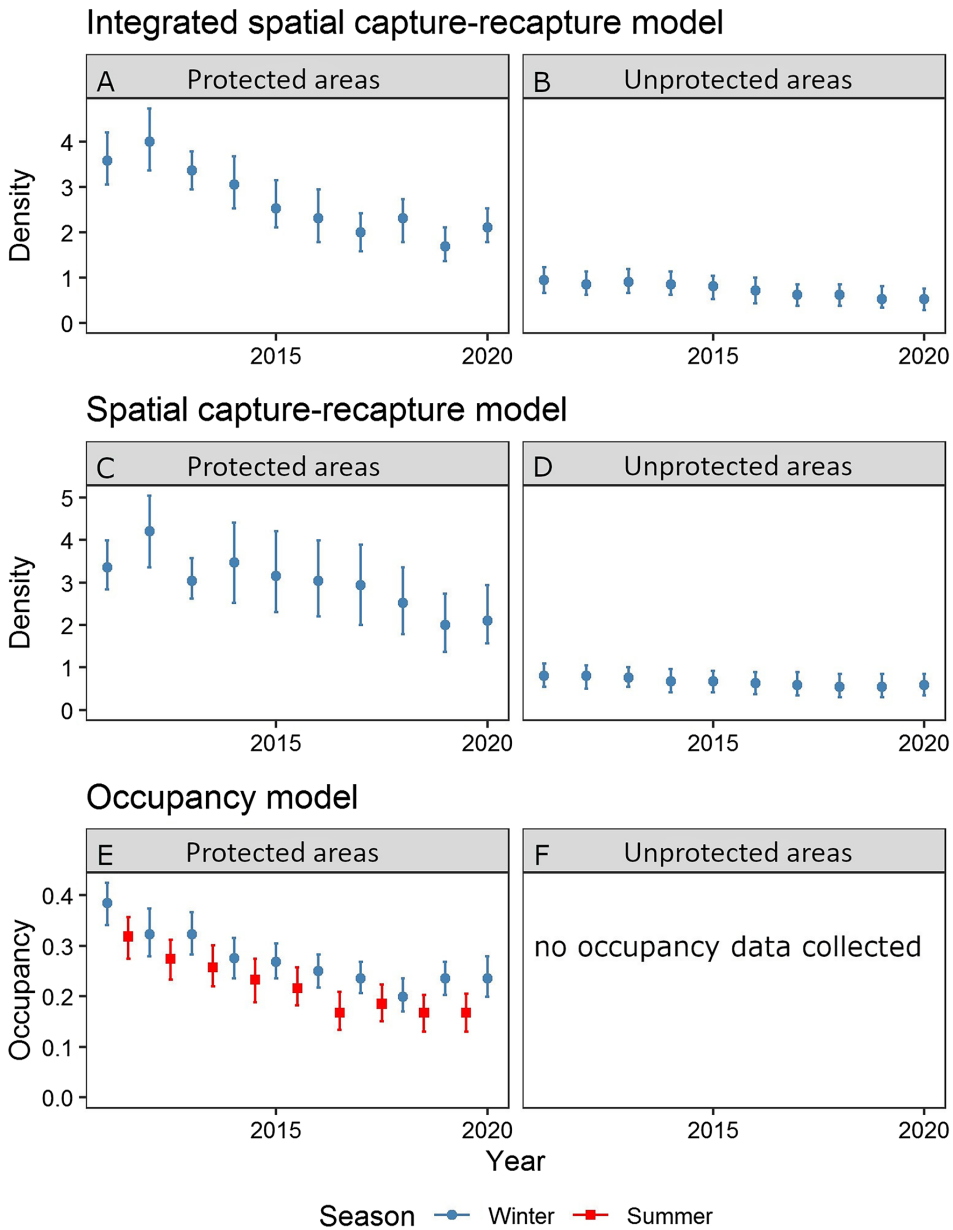
Realised wolverine (*Gulo gulo*) winter density (number of wolverines/1000 km^2^) and summer and winter occupancy estimates inside and outside of national parks from 2011 to 2020. Error bars indicate 95% Bayesian credible intervals. The integrated spatial capture-recapture model (winter; blue dots) included all detection processes including identified individuals, unknown individuals, and unmarked individuals from remote camera sampling. Density estimates are shown for the protected (**A**) and unprotected areas (**B**). The spatial capture-recapture model (winter; blue dots) included only detections of identifiable individuals, and estimates are shown for the protected (**C**) and unprotected areas (**D**). The dynamic occupancy models used summer (May–September; 2011–2019; red squares) or winter (October–April; 2011–2020; blue dots) remote camera data from broader sampling of unmarked individuals within protected areas (**E**). No occupancy data was collected for unprotected areas (**F**).

**Figure 3:**
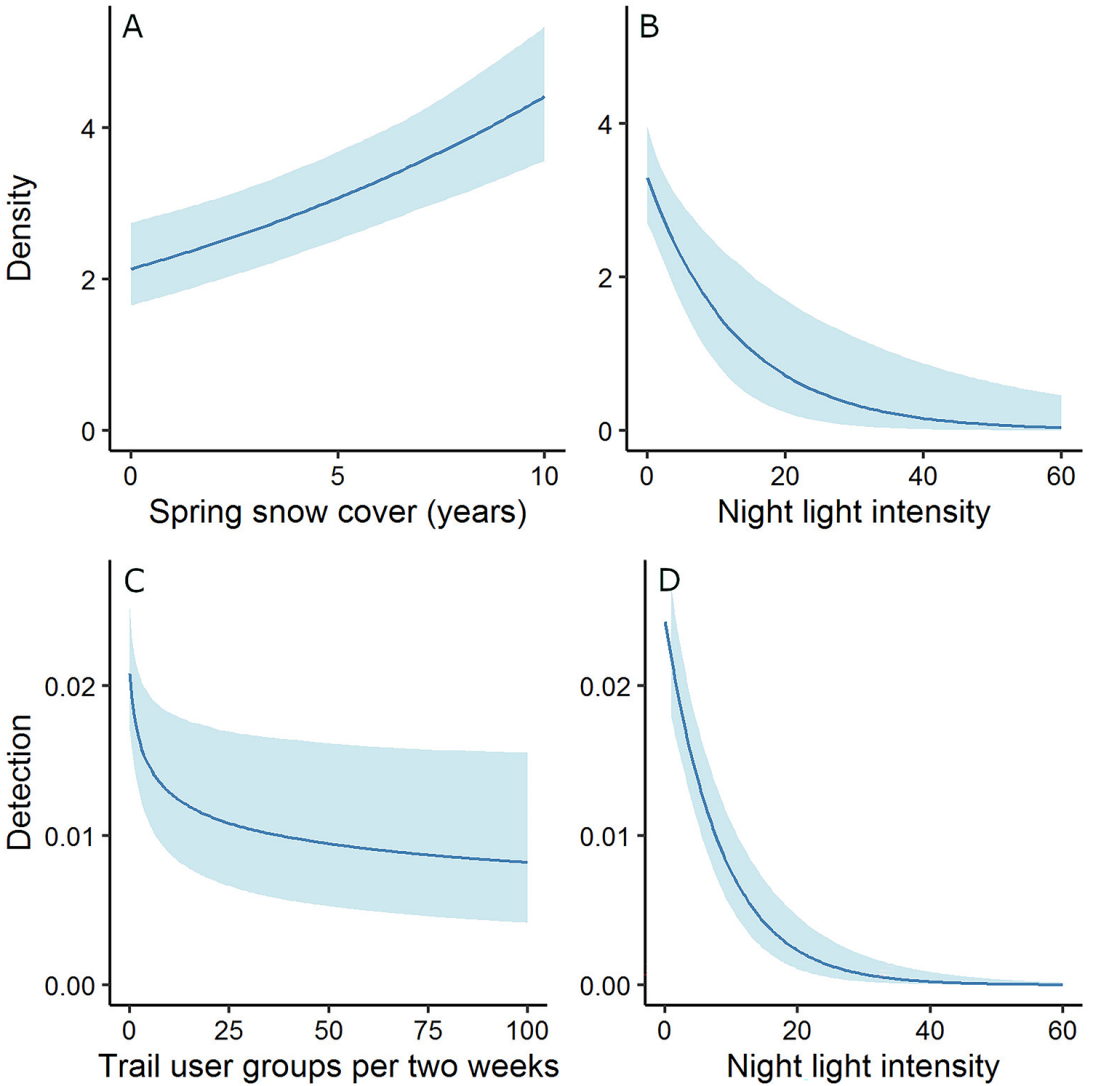
Predictions and 95% Bayesian credible intervals for the effect of spring snow cover (number of years from 2011 to 2020 with complete snow cover between 24 April and 15 May) and night light intensity on wolverine (*Gulo gulo*) density (number of wolverines/1000 km^2^), and the effects of trail use (number of trail user groups per 2o weeks) and night light intensity on wolverine detection rates.

The top model with the lowest WAIC contained density covariates for protection status, snow cover, night lights, and year, but no interaction terms and not proximity to paved roads (Supplementary Table A1). Wolverine density in 2011 was approximately 3 times higher inside than outside national parks (Fig. 2A,B, Table 2), increased with spring snow cover (Fig. 3A, Table 2), and decreased with increasing night light intensity (Fig. 3B, Table 2). Detection probability at cameras aiming at bear rub trees was higher than at other cameras (Table 2). The annual population growth rate λ was 0.924 (Table 2). Realized wolverine abundance in the study area was 54 individuals (25 females) in 2011 and 32 individuals (15 females) in 2020 (Supplementary Table A2). Realized abundance estimates inside Banff, Kootenay, and Yoho National Parks ranged from 34 individuals in 2011 (95% BCI = 29 to 40) to 20 individuals in 2020 (95% BCI’s = 17 to 24). We found no strong interactions between the population growth rate and spring snow cover, protection status, proximity to roads, or night lights. However, because of the low wolverine density, our power to detect such interactions was low.

**Table 2:** Parameter estimates from the integrated spatial capture–recapture model of a 10-year study on wolverines (*Gulo gulo*), conducted between 2011 and 2020 in the Canadian Rocky Mountains in southern Canada.

Process	Data	Parameter	Median	SD	lcl	ucl
Population	All	Lambda	0.924	0.017	0.892	0.959
Population	All	Prob male	0.559	0.060	0.441	0.680
Home range	All	SD ActivityCentre	0.001	0.000	0.001	0.002
Home range	All	Sigma female	8.998	0.417	8.235	9.824
Home range	All	Sigma male	13.248	0.568	12.192	14.426
Density	All	Intercept	− 7.105	0.100	− 7.306	− 6.912
Density	All	Night lights	− 0.295	0.099	− 0.512	− 0.128
Density	All	Park	1.327	0.069	1.191	1.464
Density	All	Snow	0.242	0.044	0.155	0.325
Density	All	Year	− 0.079	0.018	− 0.115	− 0.042
Detection	2011–2013 SCR	Intercept	− 1.270	0.142	− 1.545	− 0.983
Detection	2011–2013 SCR	Behaviour	8.756	5.566	3.351	24.183
Detection	2011–2013 SCR	Number days	0.138	0.119	− 0.089	0.379
Detection	2011–2013 SCR	Thin	0.492	0.039	0.418	0.572
Detection	2011–2020 SCR	Night lights	− 0.335	0.179	− 0.696	− 0.005
Detection	2011–2020 SCR	Snow	0.194	0.103	0.008	0.405
Detection	2018–2020 SCR	Intercept	− 0.497	0.296	− 1.046	0.111
Detection	2018–2020 SCR	Behaviour	10.548	5.420	4.316	24.247
Detection	2018–2020 SCR	Number days	0.633	0.241	0.206	1.159
Detection	2018–2020 SCR	Thin	0.407	0.042	0.331	0.493
Detection	2011–2020 Remote camera	Intercept	− 4.649	0.106	− 4.867	− 4.446
Detection	2011–2020 Remote camera	Log Trailuse	− 0.203	0.074	− 0.355	− 0.062
Detection	2011–2020 Remote camera	Night lights	− 1.089	0.175	− 1.450	− 0.762
Detection	2011–2020 Remote camera	Rubtree	0.946	0.074	0.809	1.095
Detection	2011–2020 Remote camera	Snow	0.381	0.047	0.285	0.474

Covariates are grouped according to their effect on population growth rates, home range activity centres, density, and detection processes. Covariates are then grouped by their respective source of data. *Behaviour* refers to covariates for a behavioural response at baited SCR sites, *thin* refers to probability of identifying an individual at SCR sites with marked and unmarked individuals. *number days* refers to the number of sampling days. Estimates given are the median, standard deviation (*SD*), and lower (*lcl*) and upper (*ucl*) 95% Bayesian confidence levels.

The integrated spatial capture-recapture analysis showed that realised abundance of wolverine within the study area declined by 41% from 2011 to 2020 (Fig. 2A,B). Both the simpler spatial-capture recapture analysis and dynamic occupancy models found similar declines in abundance (Fig. 2C,D), winter occupancy (Fig. 2E), and summer occupancy (Fig. 2E) (by 33%, 39%, and 47% respectively) (Supplementary Table A3). No occupancy models were developed for non-protected areas because we had few cameras there (Fig. 2F). The basic SCR model that excluded camera data produced similar but less precise population growth rates compared to the full model (λ = 0.948, 95% BCI = 0.906 to 0.900). Consistency in trends among the independent data sets adds confidence to our results showing a large decline in wolverine density. Moreover, similar to other research, our study illustrates the benefits of combining marked, unknown, and unmarked detections into integrated SCR models^21,39^, as the integrated model produced density and trend estimates with tighter confidence intervals than any of the other data sets used in isolation.

Density estimates for Banff, Kootenay, and Yoho National Parks declined from 3.6 wolverines/1000 km^2^ (95% BCI = 3.0 to 4.2) in 2011 to 2.1 wolverines/1000 km^2^ (95% BCI = 1.8 to 2.5) in 2020 (Fig. 2A). Density estimates outside of the national parks declined from 0.9 wolverines/1000 km^2^ (95% BCI = 0.7 to 1.2) in 2011 to 0.5 wolverines/1000 km^2^ (95% BCI = 0.3 to 0.8) in 2020 (Fig. 2B). Estimates of the home range scale parameter σ were 9.0 km and 13.3 km for females and males respectively (Table 2). Estimates of σ were similar to estimates from other SCR studies in the region but larger than estimates from GPS collared animals^28^.

### Responses to human activity and development

Wolverine detection probability was negatively correlated with the number of human user groups at the remote cameras. The coefficient for log(trailuse) was − 0.203 (95% BCI =  − 0.355 to − 0.062) (Table 2). Probability of detecting wolverine rapidly declined from 0 to 10 human user groups per two-week period and then more gradually declined with further increases in human activity (Fig. 3C). The confidence intervals suggested the strongest decline occurred on cameras recording no or low human use, with more variability in areas with high human use rates. Using the raw data from the winter occupancy surveys, 95% of all wolverine detections occurred in two-week periods with 3 or fewer human user groups. Night light intensity, an index without units, had a negative effect on both wolverine density (β =  − 0.295, 95% BCI =  − 0.512 to − 0.128; Fig. 3B) and detection probability (β =  − 0.335, 95% BCI =  − 0.696 to − 0.005; Table 2, Fig. 3D). The observed range of night light intensity was 0.00 to 56.634, and the 0.95 and 0.99 quantiles for detecting wolverines in a 2-week period were 0.00 and 4.04 respectively.

### Harvest rates and road mortality

Between 2010 and 2020 (10 trapping seasons), 59 wolverines were reported as harvested in the 106 traplines intersecting our study area (Supplementary Fig. A2, Table A2). Locations in the harvest records varied from precise locations to drainage or trapline. Combining British Columbia (BC) and Alberta (AB) numbers, the mean overall annual harvest rate in the study area was 13% (Supplementary Table A2). Some of these rates were likely underestimates as no harvest data was available to us for AB for 2018, 2019 and 2020, only for BC (Supplementary Table A2), and because harvest reporting compliance rates can be low^28,44^. Of the individual wolverines identified during our SCR sampling, two (1 reproductive female, detected in 2011 and 2013; and 1 male, detected in 2019) were harvested in 2015 and 2019 respectively, approximately 10 km (female) and 50 km (male) from their last SCR detection locations. During the study, 1 subadult female wolverine died in a wildlife-vehicle collision in Yoho National Park in 2012^45^.

## Discussion

Ours is one of few studies globally to quantify long-term population trends for wolverine^33^, filling a data gap that is common to other threatened species^46^. Although a large portion of our study area was formally protected, winter density and occupancy both declined by 39% over 10 years, and summer occupancy declined by 47% over 9 years. Wolverine density was 3 times higher inside versus outside protected areas, consistent with other studies in the region^28^, reflecting differences in species and landscape management inside and outside protected areas and underscoring the importance of protected areas for wolverine conservation^47,48^. However, wolverine detection probability was negatively correlated with human development and with human activity, emphasizing potential detrimental effects of recreation activities, which are often concentrated within protected areas.

Compared to other populations in North America, wolverine density was low even at the start of the study (Table S3 in Ref.^28^), and we estimated that the study area (30,689 km^2^) contained 25 females in 2011 (95% BCI: 23–27), and 15 females in 2020 (95% BCI: 13–16). In addition to human impacts, this declining population was therefore likely also increasingly influenced by demographic stochasticity whereby smaller populations require higher survival and recruitment rates to persist than larger populations^49,50^. We here discuss four possible mechanisms for the steep observed population decline, grouped into human caused mortality, non-lethal disturbances from recreation, habitat fragmentation, and food availability.

### Human-caused mortality

Transboundary animals that range across jurisdictions with differing levels of protection can face high rates of mortality^4,47,51^, and wide-ranging species are vulnerable to human-caused mortality in all but the largest protected areas^5^. For example, mortality risk for wolves (*Canis lupus*) in our study area increased 6.7 times when they travelled outside parks^52^. Wolverine have low natural adult mortality, and human caused deaths are additive to natural deaths^27,41^. For the duration of our study, wolverines were legally harvestable in the unprotected parts of our study area. The average annual harvest rate was, at ≥ 13% (Table A2), three times the 4% target rate recommended to maintain current numbers^28^. Annual rates were at or above the maximum theoretical sustainable harvest rate of 8% in all years^28^. The true average harvest rate may have been even higher, as harvest reporting compliance can be low, which makes our results conservative^28,44^. During our study, the number of wolverines outside the protected areas was very low and many animals harvested there likely originated from the protected areas. Negative transboundary effects were already apparent in 2011^36^. Harvest activity continued until 2020, which was also the last year of data analyzed here, and very likely contributed to the declining numbers we found overall, but also within the protected areas.

The legal harvest of wolverines in BC’s Kootenay-Boundary Region (~ 76,000 km^2^), which includes much of our study area, ended in 2020 based on evidence of overharvest and low regional wolverine abundance^28,53^. Our study, which contributes the first ever long-term wolverine population trend estimate in Canada, strongly supports this recent management decision. We demonstrate a declining population trend centred around protected areas which had been closed to wolverine harvest since at least 1959^35^ and were considered refugia^25^. It would thus be advisable to also examine wolverine harvest sustainability in adjacent regions, including in AB. If overharvest was the main cause of the observed population decline, population stabilization and recovery may take many years because wolverine reproductive rates are intrinsically low and affected by environmental and demographic stochasticity^54,55^. A future before-after assessment of the new harvest restrictions could help evaluate their success and help disentangle the direct effects of mortality and sub-lethal effects of humans on wolverine abundance, which is discussed in the next section.

Only one wolverine-vehicle collision was recorded in the study area during the study. Because few such road or railway deaths are documented, rates and population impacts are unknown^45^. There was no indication of poaching, which is a leading cause of wolverine mortality in Scandinavia^56^.

### Disturbances from recreation

Wolverine detection probability was strongly and negatively correlated with the number of human user groups using an area, and as few as three groups per 2-week period elicited a negative response. This pattern held for both winter and summer, and was consistent with behaviour observed in a recent study, where wolverines, particularly females, avoided habitat with high recreation intensities more strongly than those with low levels of disturbance from recreation^30^. Our study area included three of the most-visited national parks in Canada. It is possible that highly mobile species with large home ranges, such as wolverines, might incur comparatively lower costs from spatial avoidance (i.e. indirect habitat loss) than species more sensitive to increases in direct energetic expenditure^57,58^, or species with small home ranges^59^. Yet, human disturbances in many species also result in increased physiological stress and thus impact survival and reproduction^58,60–62^.

Wolverine reproductive success can be affected by habitat quality^63^, and a link has been made between levels of recreation and habitat quality^30^. In this study we did not measure fitness, which is difficult to do in wild populations of rare species. We therefore did not test if habitat avoidance due to human activity led to decreased fitness, which would indicate a strong conservation risk of human use in protected areas (Larson et al.^9^; Tablado and Jenni^10^). Nevertheless, in contrast to our first survey (2011 to 2013), between 2018 and 2020 we recaptured the same adult females each year with the potential exception of one new female, a pattern consistent with high adult survival but low recruitment, indicating that reproductive output may have decreased. Furthermore, the human use data we analyzed was from winter and spring, before weaning, when reproductive females are considered most vulnerable to disturbance^54,64,65^. In summer, after weaning but before independence, juvenile wolverines continue to depend on their mothers for food to sustain their rapid growth^64^. Levels of summer recreation in our study area are even higher than those of winter recreation. Resulting disturbances of female wolverines might impact their physical condition as well as other factors such as hunting success, which could impact their reproductive success^66^.

In conclusion, several possible causal mechanisms exist that might link the high and increasing levels of recreation in our study area to the observed declines in density and occupancy of wolverines. We used an extensive, 10-year point data set of human user group and wolverine detections and demonstrated that human activity negatively impacted wolverine detection probability at those locations. Backcountry recreation data with continuous coverage, which would allow directly measuring impact on wildlife density, are increasingly available^67,68^. They will aid the development of larger-scale quantitative spatial models of recreation, which are needed to better understand recreation’s impacts on species at risk and to inform management planning. Further research into the factors that affect female wolverine reproduction and survival is necessary to determine the causal mechanisms driving wolverine density and population trends.

### Night light intensity

Wolverine detection probability and wolverine density also declined with increasing night light intensity, which is a measure for actively used human developments. This pattern is consistent with telemetry-based findings that wolverine avoid infrastructure^69,70^. As the study population was small, habitat fragmentation and decreased population connectivity would increase the effects of demographic stochasticity, further limiting recovery potential. The impacts of human development and direct but non-lethal disturbance from recreation on population connectivity require further attention, as they may contribute to and interact with the negative effects of high-traffic roads on female wolverine connectivity^43^. Night-light intensity is available worldwide^71^, and the thresholds we identified might help researchers outline remaining suitable wolverine habitat elsewhere.

### Food availability

Density of any species is also often connected to food availability. Wolverines are opportunistic hunters and scavengers consuming a wide range of prey species; ungulate carrion is especially important^64,72^. In the adjacent Columbia Mountains, mountain caribou (*Rangifer tarandus*), mountain goat (*Oreamnos americanus*), porcupine (*Erithizon dorsatum*) and hoary marmot (*Marmota caligata*) are primary prey species for reproductive females^72^. In our study area, mountain caribou were extirpated in 2009^73^. Mountain goat populations were either stable or declining since the early 2000s (Poole^74^; S. Cherry unpublished data). Little is known about porcupine and hoary marmot populations, although porcupine may have broadly declined across British Columbia^75^. For the other ungulate species within the protected areas specifically, occupancy of elk (*Cervus elaphus*), mule deer (*Odocoileus hemionus*) and white-tailed deer (*Odocoileus virginianus*) was stable since 2011; moose (*Alces alces*) occupancy here has declined only slightly^76^, as compared to the steeper declines within British Columbia^77^. No immediate red flags about prey availability were thus apparent, but identification and better monitoring of wolverine prey species would help determine whether wolverines were limited by food availability here.

### Persistent spring snow cover

Density of wolverines was higher in areas with persistent spring snow cover. This result confirms previous findings in the region of increased density and occupancy of wolverines related to spring snow cover^28,37,78^. Several underlying mechanisms have been proposed and debated and are likely context-dependent, but the future conservation risks in light of a changing climate are not yet fully understood^26,79–81^.

## Conclusion

Our research was initiated in part because of a 2018 listing of wolverine as ‘special concern’ under the Species at Risk Act in Canada, which indicates that management information and action are required to help prevent the listed species from becoming threatened or extinct^82^. This need is exemplified by our results, which show that even within protected areas, surprisingly steep declines of species at risk can occur virtually unnoticed if key conservation concerns are not identified and addressed. Our predictions of unsustainable harvest rates outside the protected areas and of measurable negative impacts of development and recreation on wolverine density and detection probability were met. However, our analysis led us to reject our hypothesis that the protected areas acted as harvest refugia, as wolverine density and occupancy inside protected areas did not remain stable but decreased over 10 years.

Balancing outdoor recreation’s many positive aspects for humans with its increasingly evident negative impacts on species at risk will require careful collaborative management across departments and jurisdictions. Depressed population growth rates are particularly problematic for harvested species, as impacts quickly compound if harvest rates are not rapidly adjusted to account for negative trends in abundance and demographic rates. This in turn is only feasible if adequate monitoring systems are in place, which are still lacking for many species at risk and their stressors because of funding constraints and logistical challenges^33,46^. Ongoing advancements in non-invasive and large-scale survey techniques and accompanying analytical methods, including integrated spatial models of the kind we developed in this study, make such data collection increasingly practicable.

## Methods

### Study area

The study area encompassed 30,689 km^2^ of the Canadian Rockies and Purcell and Selkirk Mountains in British Columbia (BC) and Alberta (AB) including Banff, Kootenay, and Yoho National Parks and unprotected land surrounding the parks (51.2° N, 115.5° W, Fig. 1). Detailed descriptions can be found in^38,43,83^. The study area contained an internationally popular tourist destination: Official annual visitation numbers of Banff, Yoho and Kootenay National Parks grew by 29% from 4.13 million visitors in 2010 to 5.35 million visitors in 2019^84^. In winter and spring, the predominant recreational activities within the protected areas included backcountry skiing, front-country skiing, snowshoeing, ice-climbing, mountaineering, and hiking. Outside the protected areas, activities additionally included snowmobiling, cat- and heli-skiing.

### Field data collection

Four independently collected data sets were used in this analysis. The 2011–2013 study encompassed 9370 km^2^ and 80 sampling stations^36,37,43^. Not all stations were active in all years and occasions. Stations were sampled monthly (median sample interval = 31 days) between December and April. Sampling stations consisted of barbed wire wrapped around trees below bait (frozen skinned beaver) secured 2 to 3 m off the ground, and a scent lure (“Caven’s Lures—Gusto Long Distance Call”)^36^. Wolverine accessing bait left hair samples on the barbs. We only extracted and genotyped samples if a wolverine had been detected on camera at the sampling station during that occasion. Further subsampling, DNA extraction and genotyping methods are detailed in Ref.^43^. We used 7 microsatellite markers and a sex marker to differentiate individuals. Remote Covert Hyperfire and Rapidfire cameras (Reconyx, Holmen, Wisconsin) simultaneously recorded visits of wolverines at bait sites. Not all wolverine visits resulted in individual identification, because (a) some wolverine did not leave hair samples, (b) some hair samples were not analyzed, and (c) some analyzed hair samples could not be attributed to an individual^43^. Data from 2011 to 2013 for the integrated SCR model included detections of known individuals from the DNA surveys and detection/non-detection data of any wolverine, which included known and unknown individuals, from images taken by the remote cameras deployed at each sampling station (Table 1).

The 2018–2020 study encompassed 4896 km^2^ and overlapped 42% of the 2011–2013 study area (Fig. 1). Sampling occurred between December and April. Not all stations were active in all years and occasions. We used the same scent lure, bait, and sampling intervals (~ 30 days) as for the 2011–2013 survey. At each of the total 36 sites, we focussed two cameras (Reconyx Professional Covert Hyperfire I and II) on a “run pole” located below bait hung between two trees^32^. We identified wolverine with a combination of hair-based DNA sampling and photographs of their underside that showed individually unique chest markings. All genetic analyses were conducted at Wildlife Genetics International (Nelson, BC) and we used the same microsatellite markers as in the 2011–2013 survey. We classified images to individual using the pattern recognition software I^3^S Pattern+^85^, the software program Timelapse 2^86^ and a customized version of CameraBase^87^. We classified all individuals that were detected by camera or DNA, but were not identified, as unknown (Table 1).

The 2011–2020 remote camera study encompassed 13,209 km^2^ and sampled Banff, Kootenay, and Yoho National Parks with 558 cameras, many of which were part of a 10 × 10 km systematic grid designed to monitor wildlife trends (Fig. 1)^18,38^. Cameras (Reconyx Professional Covert Hyperfire and Rapidfire) were placed on hiking trails, animal trails, and near grizzly bear (*Ursus arctos*) rub trees^18,88^. We classified images using Timelapse software^86^. We divided the data into 14-day occasions and for each site and occasion we recorded detection/non-detection of wolverine.

We used wolverine kill-data collected by BC and AB provincial governments^28,78^ to estimate minimum annual trapping harvest rates. We included kills made on all trap lines intersecting our study area (Fig. A1). Details of harvest regulations and the registered trapline systems are found in Mowat et al.^28^. We obtained genetic samples from 27 of 75 wolverines trapped in the wider region between 2011 and 2020 and incorporated deaths of known individuals into our SCR models but did not include individuals in the models that were solely detected through harvest as many locations were not precise. Annual trapping seasons ended at the time our non-invasive sampling studies started, so we calculated harvest rates as $Harvest\, rate=\frac{harvested\, indivduals}{realized \,abundance+harvested \,individuals}$.

### Research and animal care permits

All applicable research and animal care permits were obtained from British Columbia provincial authorities (Wildlife Act/Collection permits CB10-68024, CB11-75845, CB12-84303 and MRCB18-284379; Park Use permits 105280 and 105895), from federal national park authorities (Parks Canada Agency Research and Collection Permit LL-2010-5652 and YNP-2018-27277), and from the University of Calgary Animal Care Committee (Certification # AC18-0112). All methods were carried out in accordance with the relevant guidelines and regulations.

### Spatial covariates

We calculated six spatial covariates: Human activity, human development, protection status, proximity to paved roads, spring snow cover, and bear rub tree. We quantified human activity by tallying the number of human user groups passing by each camera within 14-day occasions of the 2011–2020 remote camera data. We averaged number of groups across each season to reflect overall rather than finer-scale temporal effects, and log-transformed it because preliminary analysis suggested that wolverine detections decreased non-linearly with levels of human use. We pooled all types of recreation into a single metric of non-motorized recreation. We used number of groups per occasion as a covariate for detection in the integrated SCR models. We could not use this metric as a covariate for density because we lacked measures of human use on each pixel within the study area.

To calculate the human development covariate, we used NASA’s 2018 harmonized nighttime light intensity^71^. Night light intensity has several advantages over using other measures of built human footprint. Our study area included numerous areas of concentrated human activity including towns, hamlets, downhill ski areas, and outlying resorts. Night light intensity reflects intensity of human activity both where people reside and in the surrounding zone of influence with diffuse light. Night light intensity better reflects spatial variation in human activity than metrics like proximity to town or development. We included night light intensity as a density covariate and a covariate for detection probability for both SCR and remote camera data.

Protection status was determined for all sites and areas, with “protected” being defined as areas with both formal protection and no wolverine harvest (i.e., the national parks), and “unprotected” being all areas with either no formal land protection, or where wolverine harvest was permissible, or both. The areas that had formal land protection (i.e., provincial park land) but were also open to wolverine harvest, were too small to allow for a third category and were thus classified as “unprotected”.

We calculated proximity to paved roads using a decay function exp(− 0.5 × Distance to Paved Road_km_) such that values equalled 1.0 at paved roads, declined rapidly to 5 km from roads, and approached zero near 10 km. We expected wolverine to avoid areas near paved roads because some of these areas received high levels of recreational activity during winter^30^. We used proximity to paved roads as a covariate for density and included interactions between year and proximity to roads to assess how increases in visitation and recreation over time affected wolverine density.

Spring snow cover is correlated to wolverine distribution across their range^64,79^. We calculated spring snow cover using 500 m resolution moderate-resolution imaging spectroradiometer data (MODIS, dataset MYD10A1), following^79^. We calculated the number of years from 2011 to 2020 each pixel had complete snow cover between April 24 and May 15 and used a moving average with a 10 km window^28^. We included spring snow cover as a covariate for density and detection probability.

To increase detection probability of wolverine and other wildlife, we placed 15% of the remote cameras at grizzly bear rub trees^18^ and included rub trees as a binary covariate for detection probability at remote cameras. We scaled all continuous covariates by their mean and standard deviation.

### Statistical analyses

We estimated wolverine density with integrated Bayesian spatial capture-recapture models using Markov chain Monte Carlo (MCMC) techniques^17,33,89,90^. The models consisted of two hierarchical levels: one that described the underlying detection process and a second that described spatial and temporal variability in density. We defined the state-space using a 40 km buffer around the perimeter of all detector locations and discretized the resulting 30,689 km^2^ study area into 3009 pixels that had a 4 × 4 km resolution. We truncated the state space at the east side, as there is no wolverine habitat east of the Rocky Mountain Foothills (Fig. 1).

#### Detection process

We developed three different detection processes, one for each data set. For all processes, we used a half-normal detection function where the probability of detection for individual *i* at detector *j* in year *t* on occasion *k* was

$${p}_{ijtk}={{z}_{it}\lambda }_{0 ijtk} exp\left(\frac{-{Dist}^{2}}{2{\sigma }_{sex}^{2}}\right).$$

λ_0_ was the probability of detecting an individual at the home range centre. Covariates affecting λ were modelled on the logit scale. We estimated separate covariates on λ_0_ for each of the three detection processes. *Dist* was the absolute distance between animal activity centre **s**_*it*_ and detector *j* whereby *Dist*_*ij*_ =||***s***_*i*_ − **x**_*j*_||*.* We estimated a separate home range scale parameter *σ*_*sex*_ for female and male wolverine.

The integrated SCR model also included the detections of unmarked (SCR 2011–2013; Camera 2011–2020) and unknown (SCR 2018–2020) individuals to reduce bias and increase precision of density estimates (Table 1)^21,39,91^, with details in the respective detection process sections below.

##### Detection process 1: 2011–2013 DNA spatial capture–recapture

Covariates affecting λ_0_ were the sample time in days on occasion *k* and a behavioral effect for whether wolverine *i* had previously visited detector *j*, which is a strong predictor of wolverine detection^28,92^. Because some wolverine visited sites, but were not identified to individual, we included a random thinning parameter^39^, α^2011^, such that the observed probability of detection was a product of the thinning parameter (range 0 to 1) and *p*_*ijtk*_*.*

$${y}_{ijtk}^{2011}=\mathrm{Bernoulli}\left({\alpha }^{2011} {p}_{ijtk}^{2011}\right).$$

The thinning parameter was informed by remote camera detection/non-detection data collected at each site and occasion. We modelled the cumulative probability of detecting at least one individual at site *j* on occasion *k*^21,39^ as

$${p.cum}_{jtk}^{2011}=1- \prod_{i=1}^{M}\left(1- {p}_{ijtk}^{2011}\right),$$

and the likelihood as

$${y}_{ijtk}^{2011 Camera}=\mathrm{Bernoulli}\left({p.cum}_{jtk}^{2011}\right).$$

##### Detection process 2: 2018–2020 DNA and camera spatial capture–recapture

We used the same model structure and detection covariates for the 2018–2020 observation process as the SCR 2011–2013 modelling process, where

$${y}_{ijtk}^{2018}=\mathrm{Bernoulli}\left({\alpha }^{2018} {p}_{ijtk}^{2018}\right).$$

Animals were classified to individual or as unknown using both DNA and remote camera images. We used a random thinning process for unknown individuals to correct detection probabilities^39^. Unknown individuals could have been any individual from *i* = 1 to *M* including previously detected individuals (Table 1) that were detected at a rate equal to 1 − α^2018^ (the thinning parameter). We calculated the cumulative probability of detecting at least one unknown individual during occasion *k* as

$${p.cum}_{jtk}^{2018\, unknown}=1- \prod_{i=1}^{M}\left(1- {p}_{ijtk}^{2018}\left(1-{\alpha }^{2018}\right)\right),$$

and the likelihood as

$${y}_{ijtk}^{2018 \,unknown}=\mathrm{Bernoulli}\left({p.cum}_{jtk}^{2018 \,unknown}\right).$$

##### Detection process 3: 2011–2020 remote cameras

Remote cameras provided more extensive spatial and temporal sampling coverage compared to the SCR surveys and recorded the detection/non-detection of unmarked (Table 1) wolverine during each two-week occasion from 2011 through 2020. This data was formatted similarly to occupancy data. To align timing with the SCR data, for the integrated models, we only included images from October through April. Covariates for detection probability included snow cover (as above but using the 500 m pixel values at camera locations), nighttime light intensity, and the mean number of human groups per occasion. For each site, we calculated the cumulative probability of detecting at least one individual^21^, as

$${p.cum}_{jtk}^{Camera}=1- \prod_{i=1}^{M}\left(1- {p}_{ijtk}^{Camera}\right),$$

and the likelihood as

$${y}_{jtk}^{Camera}=\mathrm{Bernoulli}\left({p.cum}_{jtk}^{Camera}\right).$$

#### Population Process and trends in density

We modelled density (*D*) as an inhomogeneous point process (Royle et al.^93^, Chapter 11) where the intensity or number of wolverine activity centres μ within pixel *g* in study year *t* depended on a linear function of covariates year and snow cover such that

$$\mathrm{log}\left({\mu }_{gt}\right)= {\beta }_{0}^{D}+{\beta }_{year}^{D}\left(t-1\right)+{{\varvec{\beta}}}^{D}{{\varvec{X}}}_{g}^{D},$$

where exp(*β*_0_^*D*^) was the intercept for density in the first year of the study, ***β***^*D*^ was a vector of parameter estimates, and ***X***_*g*_^*D*^ was a matrix of spatial covariates affecting density. We chose to model changes in density as a linear function of year rather than as a dynamic model with apparent survival and recruitment because our primary metric of interest was the population growth rate $\lambda$, and $\lambda ={e}^{{\beta }_{year}^{D}}$^94^. Moreover, dynamic models contain a larger number of parameters, which we expected to be challenging to estimate because we lacked SCR data from 2014 through 2017. The expected number of individuals E(*N*_*t*_) across *G* pixels within the state space in year *t* was

$$E\left({N}_{t}\right)= \sum_{g=1}^{G}{\mu }_{gt}.$$

Using a data augmentation approach, we augmented the known population of wolverine *n* with *M*–*n* hypothetical individuals^95^. We used *M* = 100 for each year. We modelled the realised number of individuals within the state space as *N*_*t*_ ~ Binomial(*M*, ψ_*t*_), where ψ_*t*_ was the inclusion probability for whether individual *i* was included in the population during year *t*. The inclusion parameter ψ_t_ for whether known or hypothetical individual *z*_*it*_ was alive was calculated as *E*(*N*_*t*_)/*M*. We modelled the latent inclusion state of individual *i* as *z*_*it*_ ~ Bernoulli(ψ_*t*_). We fixed *z*_*it*_ to equal 1 between the first and last year individual wolverines were detected and fixed *z*_*it*_ to equal 0 after individuals had been harvested. We included sex-specific estimates for the home range scale parameter σ (see below). We modelled the sex of known and hypothetical individuals as *z*_*i*_ = Bernoulli(*p*_*male*_) where *p*_*male*_ was the probability that an individual was male.

Activity centers **s**_*it*_ in year one were uniformly distributed across the state space. The probability of an activity centre **s**_*it*_ occurring in pixel *g* was π_*gt*_ = μ_*gt*_/*E*(*N*_*t*_), which we modelled as a categorical random variable across all pixels *G*,

$${s}_{it}\sim \mathrm{Categorical}\left({\pi }_{1t}\dots {\pi }_{Gt}\right).$$

Activity centre locations in years two to 10 depended on both μ_*gt*_ and a Gaussian random walk from their previous activity center^33,96^ where

$${{\varvec{s}}}_{it}\sim \mathrm{Normal}\left({{\varvec{s}}}_{i,t-1},{\sigma }_{s}^{2}I\right).$$

The parameter σ_s_^2^ describes the variation in distance moved from the previous activity center and *I* is the identity matrix for movement in both cardinal directions.

We calculated realized abundance estimates across the state-space for each year as, ${\widehat{N}}_{t}= \sum_{i=1}^{M}{z}_{it}$. We tallied realised abundance and density estimates within and outside national parks using the posterior MCMC samples of *z*_*it*_ and activity center locations **s**_*it*_.

#### Comparison of integrated SCR and SCR models

Density covariates in our baseline integrated SCR model included the effects of year, protection status (inside versus outside of national parks), and snow cover^28^. We compared our baseline model to ten other models (Table A1) that included the covariates proximity to roads, night light intensity, and interactions between (a) year and protection status because we expected wolverine densities to be higher in protected areas and remain stable over time there; (b) year and proximity to roads as well as year and night light intensity to assess effects of increasing visitation; and (c) year and snow cover to assess potential changes in habitat conditions on population growth rates. We then selected the model with the lowest WAIC^97^.

We assessed the relative influence of each set of data used in the integrated SCR model on population trend. Our integrated SCR model contained spatial capture-recapture data, unmarked and unknown individuals at SCR sites, and unmarked individuals from the remote cameras. We compared the integrated SCR model with a simple SCR model that restricted the analysis to identified individuals and excluded thinning data and the remote camera data.

#### Winter and summer dynamic occupancy models

Finally, we used remote camera data to develop dynamic occupancy models^98^ that quantified changes in occupancy from 2011 to 2020. We created separate occupancy models for summer and winter. The dynamic occupancy models contained covariates for occupancy (snow cover) and detection probability (snow cover, number of human groups per occasion, and rub trees). We compared models with and without the effects of snow cover and year on colonization and extinction. We estimated trends using realized occupancy estimates and the population growth rate λ as the change in realized occupancy estimates from 2011 to 2020.

We used weak Normal(0, SD = 10) priors for the home range scale parameter ln(σ_sex_), the Gaussian random walk movement parameter for activity centers ln(σ_*s*_), and for all detection covariates on the logit scale. We sampled each of three MCMC chains for 20,000 iterations, discarded the first 5000 iterations as a burn-in, and thinned the data to every 10th iteration. We reported the 95% Bayesian credible interval (BCI). We estimated model parameters using R version 4.0.5^99^ and nimble version 0.11^100^. We provide our model, written in BUGS language in Supplementary Appendix B and on the Dryad Digital Repository 10.5061/dryad.z34tmpghh^101^.

## Supplementary Information

Supplementary Information 1.

Supplementary Information 2.

## Data Availability

Data and R scripts for running the integrated SCR model are available from the Dryad Digital Repository 10.5061/dryad.z34tmpghh^101^.
