# Supplementary material for: Protection status, human disturbance, snow cover and trapping drive density of a declining wolverine population in the Canadian Rocky Mountains
Source: Sci Rep. 2022 Oct 24;12:17412. doi: 10.1038/s41598-022-21499-4 (PMC9592595; doi:10.1038/s41598-022-21499-4)
Supplement: Supplementary file 1 — Supplementary Information 1. [file 41598_2022_21499_MOESM1_ESM.docx]

# Supplemental Materials

Appendix A – Supplementary Figures and Tables

Figure A1. Number of wolverines (Gulo gulo) reported to have been harvested (n=59) in each registered trapline intersecting our study area (30 689 km^2^) between 2010 to 2020. Only traplines that intersect the study area are outlined, with 67 traplines in British Columbia and 39 in Alberta. Sources for the base map include Esri, Airbus DS, USGS, NGA, Nasa, CGIAR, N Robinson, NCEAS, NLS, OS, NMA, Geodatastyrelsen, Rijkswaterstaat, GSA, Geoland, FEMA, Intermap, and the GIS user community. This map contains information licensed under the Open Government Licence – British Columbia (https://www2.gov.bc. ca/gov/content/data/open-data/open-government-licence-bc), the Open Government Licence – Alberta (<https://open.alberta.ca/licence>) and the Open Government Licence – Canada (https://open.canada.ca/en/open-government-licence-canada) and was created in ArcMap 10.7.1 (<https://support.esri.com/en/products/desktop/arcgis-desktop/arcmap/10-7>).


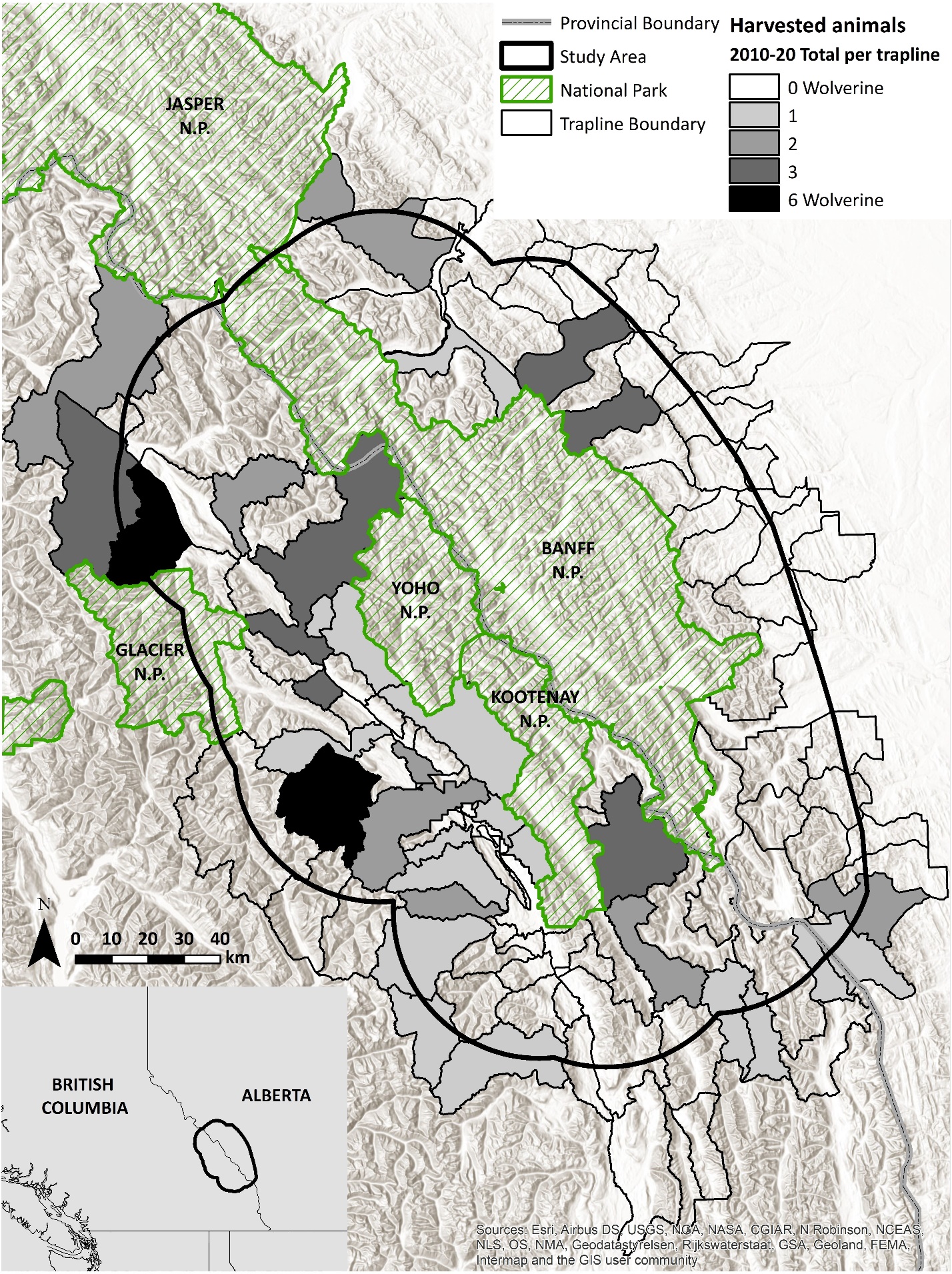


Table A1 Comparison of integrated spatial capture-recapture models of wolverine (*Gulo gulo*) based on WAIC. Models assessed the effects of protection status (Park), persistent spring snow cover (Snow), and distance from paved roads (NearRoad) on density.

| Model | WAIC | dWAIC | Comments |
| --- | --- | --- | --- |
| Year + Park + Snow + NightLight | 20675.9 | 0.0 |  |
| Year + Park + Snow + NightLight + Year:Snow | 20676.8 | 0.9 | 90% BCI for interaction term overlapped 0 |
| Year + Park + Snow + NightLight + Year:Nightlight | 20677.7 | 1.7 | 90% BCI for interaction term overlapped 0 |
| Year + Park + Snow + NightLight + Year:Park | 20678.2 | 2.2 | 90% BCI for interaction term overlapped 0 |
| Year + Park + Snow | 20685.7 | 9.8 |  |
| Year + Park + Snow + Year:Snow | 20689.1 | 13.2 | 90% BCI for interaction term overlapped 0 |
| Year + Park + Snow + Year:Park | 20689.5 | 13.6 | 90% BCI for interaction term overlapped 0 |
| Year + Park + Snow + NearRoad + Year:Snow | 20690.1 | 14.2 | 90% BCI for interaction term overlapped 0 |
| Year + Park + Snow + NearRoad + Year:Park | 20690.4 | 14.5 | 90% BCI for interaction term overlapped 0 |
| Year + Park + Snow + NearRoad + Year:NearRoad | 20690.7 | 14.7 | 90% BCI for interaction term overlapped 0 |
| Year + Park + Snow + NearRoad | 20695.9 | 20.0 |  |

Table A2 Annual numbers of wolverines (*Gulo* *gulo*) harvested in the British Columbia (Harvested BC) and Alberta (Harvested AB) parts of the study area; realized wolverine abundance for the study area estimated with integrated spatial capture—recapture models (R. A. study area), minimum harvest rates (H. R. study area) for the study area; realized wolverine abundance for the non-protected (harvested) areas only (R. A. non-protected); minimum harvest rate for the non-protected (H. R. hon-protected) areas; by trapping season (Trapping season). Reflected are individual wolverines reported trapped within all British Columbia (n=67) and Alberta (n=39) traplines that intersected the study area. The number of harvested individuals in Alberta traplines was not available for 2017/18, 2018/19, and 2019/20 trapping seasons, therefore the presented harvest rates for those years may be an underestimate. Study area size was 30 689 km^2^.

| *Trapping season* | *Harvested BC* | *Harvested AB* | *R. A.*  *study*  *area* | *H. R.*  *study*  *area* | *R. A.*  *non-protected* | *H. R.*  *non-protected* |
| --- | --- | --- | --- | --- | --- | --- |
| 2010/11 | 3 | 2 | 54 | 8% | 20 | 20% |
| 2011/12 | 4 | 1 | 56 | 8% | 18 | 22% |
| 2012/13 | 6 | 3 | 51 | 15% | 19 | 32% |
| 2013/14 | 3 | 1 | 47 | 8% | 18 | 18% |
| 2014/15 | 2 | 3 | 41 | 11% | 17 | 23% |
| 2015/16 | 8 | 0 | 37 | 18% | 15 | 35% |
| 2016/17 | 2 | 2 | 32 | 11% | 13 | 24% |
| 2017/18 | 4 | - | 34 | 11% | 13 | 24% |
| 2018/19 | 9 | - | 28 | 24% | 11 | 45% |
| 2019/20 | 6 | - | 32 | 16% | 11 | 35% |
| MEAN | 4.7 | 1.2 | 41.2 | 13% | 15.5 | 28% |

Table A3 Parameter estimates from a dynamic occupancy model for wolverine (*Gulo gulo*) using winter (October – April) data. Data was collected using remote cameras from 2011 to 2020 in Banff, Yoho and Kootenay National Parks in British Columbia and Alberta, Canada.

| *Process* | *Parameter* | *Estimate* | *SE* | *z* | *P-value* |
| --- | --- | --- | --- | --- | --- |
| Occupancy | Intercept | -0.676 | 0.550 | -1.228 | 0.219 |
| Occupancy | SnowCover 10km | 1.332 | 0.382 | 3.492 | 0.000 |
| Occupancy | NightLights | -1.207 | 2.066 | -0.584 | 0.559 |
| Colonization | Intercept | -2.570 | 0.175 | -14.718 | 0.000 |
| Extinction | Intercept | -1.110 | 0.178 | -6.225 | 0.000 |
| Detection | Intercept | -2.411 | 0.094 | -25.608 | 0.000 |
| Detection | RubTree | 0.261 | 0.092 | 2.832 | 0.005 |
| Detection | SnowCover 500m | 0.163 | 0.064 | 2.533 | 0.011 |
| Detection | NightLights | -0.854 | 0.214 | -3.986 | 0.000 |
| Detection | log(NumberOfPeopleEvents) | -0.103 | 0.073 | -1.409 | 0.159 |

Table A4 Parameter estimates from a dynamic occupancy model for wolverine (*Gulo gulo*) using summer (May – September) data. Data was collected using remote cameras from 2011 to 2019 in Banff, Yoho and Kootenay National Parks in British Columbia and Alberta, Canada.

| *Process* | *Parameter* | *Estimate* | *SE* | *z* | *P...z..* |
| --- | --- | --- | --- | --- | --- |
| Occupancy | Intercept | -1.241 | 1.042 | -1.191 | 0.234 |
| Occupancy | SnowCover 10km | 1.163 | 0.714 | 1.628 | 0.103 |
| Occupancy | NightLights | 2.860 | 3.266 | 0.876 | 0.381 |
| Colonization | Intercept | -2.888 | 0.450 | -6.416 | 0.000 |
| Extinction | Intercept | -0.996 | 0.367 | -2.718 | 0.007 |
| Detection | Intercept | -3.120 | 0.260 | -12.017 | 0.000 |
| Detection | RubTree | 0.624 | 0.185 | 3.379 | 0.001 |
| Detection | SnowCover 500m | 0.785 | 0.252 | 3.120 | 0.002 |
| Detection | NightLights | -1.537 | 0.487 | -3.156 | 0.002 |
| Detection | log(NumberOfPeopleEvents) | -0.111 | 0.066 | -1.679 | 0.093 |

Table A5 Occupancy trend estimates for wolverine (*Gulo gulo*) using a dynamic occupancy model on data collected in summer (May – September). Data was collected using remote cameras from 2011 to 2019 in Banff, Yoho and Kootenay National Parks in British Columbia and Alberta, Canada.

| *Year* | *Estimate* | *lcl* | *ucl* |
| --- | --- | --- | --- |
| 2011 | 0.318 | 0.274 | 0.356 |
| 2012 | 0.274 | 0.233 | 0.312 |
| 2013 | 0.257 | 0.219 | 0.301 |
| 2014 | 0.233 | 0.188 | 0.274 |
| 2015 | 0.216 | 0.182 | 0.257 |
| 2016 | 0.168 | 0.134 | 0.209 |
| 2017 | 0.185 | 0.151 | 0.223 |
| 2018 | 0.168 | 0.130 | 0.202 |
| 2019 | 0.168 | 0.130 | 0.205 |
